# Development of a scale for determining violence against infertile women: a scale development study

**DOI:** 10.1186/1742-4755-11-18

**Published:** 2014-02-28

**Authors:** Güliz Onat

**Affiliations:** 1 Health Sciences Faculty, Nursing Department, Istanbul Aydın University, Beşyol Mah. İnönü Cad. No: 38, Küçükçekmece, Istanbul, Turkey

**Keywords:** Infertility, Scale development, Violence, Infertilité, Développement à grande échelle, La violence

## Abstract

**Background:**

To develop a scale to evaluate violence experienced among infertile women.

**Method:**

Three steps were followed in the development of the scale: Literature review and deep interviews to generate item pool, content validity testing, and administration of draft. Content validity was evaluated by experts. The draft scale was pilot-tested with a convenience sample of 30 women during their treatment. After the pilot-test, 166 infertile females filled the scale in the infertility clinic of a university hospital in Istanbul.

**Results:**

For evaluation of construct validity, Kaiser-Mayer Olkin was 0.91. Bartlett test was statistically significant (p = 0.00). According to the results of analysis, 5 domains were determined: “domestic violence”, “social pressure”, “punishment”, “exposure to traditional practices” and “exclusion”. The values of correlation of item were between 0.50 and 0.82. Item-total and subscale-total correlation varied between 0.57-0.91. The scale had good internal reliability, with Cronbach’s Alpha coefficient of 0.96. The other coefficients of subscales varied between 0.80-0.94.

**Conclusions:**

The scale called “Infertile Women’s Exposure to Violence Determination Scale” indicates high reliability, good content and construct validity. Routine screening for domestic violence in infertility clinics is necessary to give affected women an opportunity to access appropriate health care and support services. On the other hand, common use of Infertile Women’s Exposure to Violence Determination Scale in infertility clinics provides increased sensitivity and awareness by caregivers.

## Abstract (fr)

**Abstrait:**

**Fond**

d’élaborer une échelle pour évaluer la violence expérimenté parmi les femmes infertiles.

**Méthode**

Trois étapes ont été suivies dans le développement de l’échelle: Revue de la littérature et des interviews profondes pour générer piscine point, les tests de validité du contenu, et l’administration du projet. La validité de contenu a été évaluée par des experts. Le projet de barème a été mis à l’essai auprès d’un échantillon de commodité de 30 femmes au cours de leur traitement. Après un essai pilote, 166 femmes infertiles remplies à l’échelle clinique d’infertilité d’un hôpital universitaire à Istanbul.

**Résultat**

Pour l’évaluation de la validité de construction Kaiser-Mayer Olkin était de 0,91. Bartlett test était statistiquement significative (p = 0.00). Selon les résultats de l’analyse, 5 domaines ont été déterminés: «violence domestique», «pression sociale», «punition», «l’exposition à des pratiques traditionnelles “ et “exclusion”. Les valeurs de corrélation de point situaient entre 0,50 et 0,82. Corrélation élément-total et sous-échelle-total ont varié entre 0,57-0,91. L’échelle a une bonne fiabilité interne, avec le coefficient alpha de Cronbach de 0,96. Les autres coefficients de sous-échelles ont été modifiées entre 0,80-0,94.

L’échelle qui appelle “l’exposition de femmes infertiles à la violence échelle de détermination” est un gage de fiabilité, le bon contenu et la validité. Le dépistage systématique de la violence domestique dans les cliniques de fertilité est nécessaire de donner aux femmes touchées par la possibilité d’accéder aux soins de santé appropriés et des services de soutien. D’autre part, l’utilisation commune de l’exposition de femmes infertiles à la violence échelle de détermination dans les cliniques de fertilité offre une sensibilité accrue et la sensibilisation des soignants.

## Introduction

Infertility is generally defined as the inability to conceive after 12 months of regular unprotected sexual intercourse. Infertility is a life crisis because of its uncertain and individual outcomes [1]. Infertility affects 10-15% of all couples in the United States [2-6]. With its emotionally threatening and stressful nature and high cost, infertility is a life crisis for both men and women. It is not only a gynaecological illness but also a bio-psycho-social health problem including a lower quality of life (QoL), psychiatric problems, marital conflicts and sexual dissatisfaction [7,8]. Stigmatization, loss of potency, role failure, and reduced self-esteem are negative results of infertility. Feelings of personal and sexual inadequacy, sexual dysfunction, depression, anxiety, hostility, and guilt have been reported [9].

Domestic violence is a public health problem, which threatens physical and mental health considerably worldwide. Domestic violence mostly occurs in family environments and against women. It is reported that one in three women are exposed to physical or sexual violence by the men in their lives [10]. Especially in patriarchal societies, if a woman cannot bear, she might be exposed to violence in various ways [11,12]. Unisa [11] and Dyer et al. [12] showed that women got various punishments in their societies [11,12]. In Unisa’s study, which is conducted on 316 childless women, 39% of the women reported that they have been exposed to violence by their husbands, 4% of their husbands had one more relationship, 12% had more than one relationship, and 4% wanted divorce [11]. Negative reactions from the people around an infertile person are an effective factor that might lead to deterioration of the health of the infertile person. The person might be exposed to psychological violence via social isolation, stigma, humiliating curious questions, and pressure from his/her family [11,12].

To solve problems related to domestic violence, most major medical organizations (including the American Medical Association [AMA], the American Academy of Pediatrics [AAP], the American Academy of Family Physicians, the American College of Obstetricians and Gynecologists, and the American College of Emergency Physicians) recommend routine intimate partner violence screening as part of standard patient care [13]. There are many studies that include developing tools such as “Hurt, Insult, Threaten, and Scream” (HITS), “Abuse Assessment Screen” (AAS) and Partner Violence Screen (PVS) to screen abuse or violence among women by health care providers [13]. Some studies showed models to understand the nature of violence and then develop coping strategies [14,15]. At the end of the literature screening, -although there are some studies which were conducted by tools in order to measure stress levels associated with being infertile; such as the Fertilize Problem Inventory (FSE) [16]- unfortunately, no comprehensive study that evaluates violence in infertility with all of its aspects (physical, emotional, economical, and sexual) has been reached. It has been noted that the lack of studies related to infertility and violence is caused by the lack of a tool to specifically evaluate the violence in infertility. The lack of a tool is a major gap. The contribution of the study related to its implications for practice is that professionals can use it to scan women who were exposed to violence for having involuntary childlessness. They can use it in routine patient care and easily detect which patients need to be involved in a consultation program. After cumulative data, a guide can be developed to prevent violence among infertile couples. On the other hand, common use of this scale in infertility clinics provides increased sensitivity and awareness of caregivers. The contribution of the development of such a scale for policy-maker decisions will be that, thanks to this scale, detection of violence among infertile couples will be possible. The data collected by this scale might give clues to develop and consult policies. The contribution of the study related to future researches is that, conducting studies related to infertility and violence will be possible thanks to this scale. Cultural and lingual validities can make it possible to use it in other societies. This scale can set an example for the development of future tools for infertile couples, including men.

The aim of this study is to develop a tool that can be used by health professionals as a diagnostic tool in order to evaluate violence against infertile women.

## Methods

### Participants

The participants were chosen among people who had treatment between October 2009 - June 2011 in Istanbul University, Istanbul Medical School, Division of Reproductive Endocrinology and Infertility. A total of 200 infertile males and females were invited to be participants of the study. The selection criteria were: being primary infertile (i); being diagnosed with infertility but not under treatment (ii). The patients who are not under any treatment were chosen in order to minimize the effects of treatment distress on answers. Because it is known that while taking a medication, patients’ answers might be affected by distress. They were called on the phone. The objective of the study was explained; guarantee was given for privacy of answers and interview settings were explained. They were invited to be a participant of the study. Only 166 females and 132 males wanted to volunteer. Response rates were 83% for females, 61% for males. According to statistical analyses, responses of the males were not convenient for analysis because of being monotonous. For this reason, males were excluded from the study. So, this study was conducted on 166 infertile women only.

### Information of the clinic settings

This study was conducted at Istanbul University, Istanbul Medical School, Division of Reproductive Endocrinology and Infertility. Twenty patients are examined in this clinic every day. It takes at least six months to get a certain diagnosis for infertility. If there is no medical indication for in-vitro fertilization (IVF), the female takes three interventions (intrauterine insemination) before an IVF attempt. The cost of the treatment, depending on the type of treatment, is between about 500–2000 Euros.

### Sample size

To calculate the sample size in scale development studies, it is often suggested that five to ten subjects be included per item, depending on the number of items in the draft scale [17]. Therefore, a total of 166 cases were considered adequate to perform reliability/validity analyses since the scale included 31 items (31×5 = 155). According to another commonly used approach, the sample size should be adequate to perform statistical procedures such as factor analyses; a sample of 100 is classified as poor, 200 as fair, 300 as good, 500 as very good and 1000 as excellent. However, a sample size of 200 is adequate in most cases for ordinary factor analysis that involves around 40 items [18,19].

### Procedures followed for scale development and analyses

Three steps were followed in development of the scale: Literature review and deep interviews to generate item pool, content validity testing, administration of draft.

### Literature review and deep interviews to generate item pool

In the first stage, literature was comprehensively scanned. In order to generate item pool; two books, one report, ten doctorate dissertations and twenty-two articles on violence in infertility, four statistics books and two articles on developing a tool were read. Some of these have been cited in the reference section. Two forms were developed by the researchers at the end of the literature scan. Form I had 28 questions, which dealt with socio-demographic characteristics, stories of marriage and infertility. Form II had several open-ended questions on both violence exposed to physically, emotionally, economically, sexually and social pressure such as isolation and stigma. In order to generate item pool, 16 infertile males and females were interviewed as individual in-depth by using these forms. These 16 infertile people were chosen among those who have had a treatment in the mentioned clinic by scanning patient files. Individual interviews were preferred over a focus group due to the nature of the issue. It was necessary to consider privacy when talking about violence.

To obtain reliable data;

❑ The interviews were conducted by the same researcher (GO) in a private room,

❑ The researcher attended a training program on qualitative research methods before she conducted the in-depth interviews,

❑ The researcher did not conduct more than two interviews in a day,

❑ To minimize recall-bias, the researcher prepared a report for every interview and the interview data were transcribed at the end of the day it was conducted,

❑ Participants were informed that the researcher used a tape recorder to collect data and they were asked for permission,

### Content validity testing

At the end of the in-depth interviews, a draft scale was prepared with 38 likert items. The items were prepared as “all the time, generally, sometime, rarely, never” consecutively. “Never” was 1 point, “all the time” was 5 points. The draft scale was sent to eight experts (one psychiatrist, one clinic psychologist, six midwifery and nursing faculty academics specialising in obstetrics) to collect their suggestions. The content validity index (CVI) is a widely used index that provides evidence for content validity by using ratings of item relevance by a panel of content experts. Experts rate each item as: 1, not relevant; 2, somewhat relevant; 3, quite relevant; 4, highly relevant. Ratings for either 3 or 4 are considered to be relevant. Agreement for relevance at the item level should be at least 80% (e.g. eight out of 10 experts should rate 3 or 4 to have an item CVI score of 0.80). The average item CVI score is the average CVI score of the scale [17,20,21]. For the expert reviews, a minimum of three experts is advised, but more than 10 is probably unnecessary [17]. In our study at least six of eight experts’ ratings must be 3 or 4 required for a minimum CVI item score of 6/8 = 0.75 for each item. After the revisions suggested by the experts, the scale was re-arranged as 31 items. The draft scale was pilot-tested with a convenience sample of 30 women during their treatment in the abovementioned infertility clinic. Approximately fifty patients were called for invitation to the study. After reaching the first thirty patients who wanted to volunteer, we stopped calling and those thirty patients were taken in for a pilot study. The selection criteria was the same as the sampling group: being primary infertile (i); being diagnosed with infertility but not being under treatment (ii).

### Data collection

The scale with 31 items was applied to 298 infertile females (166) and males (132). They filled up the scale by themselves in a private room when they were waiting for examination on the appointment day. The duration of filling up the scale was approximately 10–15 minutes.

### Data analysis

The items of the scale varied from 1-never, to 5-all the time. The total score was calculated by adding up points from each item. The maximum score was 155 and the minimum score was 31. Higher scores mean that exposure to violence is more frequent. In order to evaluate separately by gender (male and female), the database was split. All statistical analyses were made separately for each gender. The value of Kaiser-Meyer-Olkin was 0.91 and Bartlett’s 464 (p = 0.00) for females; 0.66 and 465 (p = 0.00) for males. In the entire group, Kaiser-Meyer-Olkin was 0.92, Bartlett’s was 465 (p = 0.00). The Kaiser-Meyer-Olkin of males was less. For this reason, males were excluded from the study and analysis.

The Statistical Package for Social Sciences (SPSS) 11.0 for Windows was used to analyse the data. Descriptive statistics, factor analyses and Cronbach alpha test were used. The statistical significance level for confidence was taken as 95% and P values as 0.05.

### Ethical consideration

The participants were recruited on a voluntary basis and all of them were informed about the objectives of the study as well as the confidentiality of the data. Informed consents were taken from them. This project was approved by the Research Ethics Committee of the hospital.

## Results

### Characteristics of the participants

The mean age was 29.96 ± 4.77. The duration of education was 6.82 ± 3.49 years. 74.7% of women were housewives. 42.2% women reported that their expenses were more than their income. 72.3% have nuclear family. The findings related to marriage story: age of marriage was 22.22 ± 4.57; number of marriages was 1.09 ± 0.50; duration of marriage was 7.79 ± 4.83 years. The findings related to infertility story: the duration of infertility was 7.09 ± 4.80 years and treatment was 3.49 ± 3.40 years. 33.7% have female factor infertility, 30.1% have male factor, 7.8% have mixed type and 28.3% have unexplained infertility.

### Construct validity: the factor analyses

Factor analysis is a useful analytical tool that can identify potential underlying dimensions/subscales in a scale [17-19,22]. The exploratory factor analysis was used for content validity. Principal Components Analysis and varimax rotation were used in order to detect extraction of factors (Table  1). Kaiser-Mayer Olkin was 0.91. Bartlett test was statistically significant (p = 0.00).

**Table 1:** Factor load of the final form of the IWEVDS’s subscale-total score and Cronbach’s alpha

**Items**	**1**	**2**	**3**	**4**	**5**
My partner threatens me with divorce because I am childless.	0.82				
My partner abstains from kissing and touching me because I am childless.	0.80				
My family abstains from giving me property rights because my disability hinders the reproduction of my family.	0.77				
My partner thinks on getting married to a fertile woman since I can’t have children.	0.76				
My partner abstains from having sex with me because I am childless.	0.75				
I am exposed to kicks, fists, and slaps because I am childless.	0.70				
My partner abstains from visiting his family together with me because I am childless.	0.67				
My partner does not say words of love to me because I am childless.	0.58				
My partner says humiliating words to me concerning my sexual performance because I am childless (incapable, frigid etc.)	0.58				
I sometimes get humiliated in front of others because I am childless.	0.57				
Despite my unwillingness, I get insisting requests to visit some relatives who have children.	0.55				
I am excluded by people around me because I am childless.		0.77			
I am not greeted because I am childless.		0.72			
I am pointed out as “the childless woman”		0.66			
People gossip about my childlessness.		0.61			
People around me consider me as disabled and guilty.		0.53			
Because of my inability to reproduce, comments concerning my womanhood, which saddens me, are made.		0.52			
People around me blame me all the time because I am childless.		0.50			
I am exposed to heavy punishment such as tough housework because I am childless.			0.68		
Any kind of failure that I have is associated with my disability to bear a child.			0.66		
People give me nicknames related to my disability to bear (infertile, castrated, unproductive etc.)			0.58		
People sometimes do not invite me to family reunions that include children.			0.58		
Despite my unwillingness, my partner insists on having sexual intercourse in order to conceive all the time.			0.54		
Even though I am not the reason for the infertility, I am under pressure to tell other people that the disability belongs to me.			0.52		
I am exposed to curious questions such as “When will you give a birth?”				0.78	
In order not to be exposed to curious questions, I have to tell lies or give evasive answers.				0.78	
Despite my unwillingness, I am forced to eat some food which is believed to facilitate conception (honey, hazelnuts, restoratives, ram’s testicles)				0.70	
Despite my unwillingness, I am exposed to several traditional practices which are thought facilitate conception (imam prays for a couple in order to break a spell etc.)				0.54	
I am compared to fertile women all the time.					0.66
I am held responsible for/being accused of any random misfortune in life because I am childless.					0.56
I am not allowed in decision-making mechanisms in my family.					0.52
**The rates of explanations of variants (%)**	**23.6**	**15.2**	**13.1**	**12.4**	**7.7**
**Croncbach’s alpha coefficient**	**0.94**	**0.89**	**0.91**	**0.81**	**0.80**

### Subscale analyses

As an indicator of internal consistency, each subscale extracted from factor analysis was evaluated in terms of its correlation with the total scale as well as the item-subscale correlation. A higher correlation coefficient indicates a stronger relationship with the item and the nature of content intended to be measured. Correlations > 0.25-0.30 and < 0.70 are preferred [17]. In our study, 0.30 was taken as the lower limit for item-total correlations. The correlation coefficients are shown in Table  1. The values of correlation of item were between 0.50 and 0.82.

In order to determine sub-scales, factor analysis was made on females’ data. According to the results of the analysis, 5 domains were determined.

1. Domestic violence domain; which consists of 11 items related to physical, economical, emotional, sexual violence and marital difficulties such as threat of divorce, consideration of marrying a fertile partner, humiliation, not presenting affection (items 3, 4, 7, 8, 9, 10, 11, 12, 14, 22, 30).

2. Social pressure domain; which consists of 7 items related to social difficulties such as stigma, isolation, humiliation, gossip, being made to feel guilty and disabled by community (items 1, 2, 6, 15, 19, 20, 21).

3. Punishment domain; which consists of several areas (6 items) related to insistence on sexual intercourse, nicknaming, being subjected to exhausting housework, not being invited to houses of relatives/neighbours, being charged with inability and being forced to own the infertility’s cause (item 13, 17, 27, 28, 29, 31).

4. Exposure to traditional practices domain; which consists of several areas (4 items). Despite an infertile woman’s unwillingness, forcing her to eat some kind of food which is believed to facilitate conception (item 23), going to places to reverse a spell (item 24), being exposed to curious questions about having a child (item 25) making them tell a lie or give an evasive answer (item 26).

5. Exclusion domain; which consists of 3 items: being held responsible/being accused of any random misfortune in life because of being infertile, not being allowed in decision-making mechanisms, being compared to fertile women all the time (item 18).

### Internal reliability

Cronbach’s alpha coefficient is about the degree of inter-relatedness between a set of items designed to measure a single construct. A reliability coefficient of 0.70 may be sufficient for a new scale, but it is expected to exceed 0.80 for a mature scale [17-19,22]. In this study, Cronbach’s alpha coefficient was 0.96. The other coefficients of subscales were: 0.94 for domestic violence domain; 0.89 for social pressure domain; 0.91 for punishment domain; 0.81 for exposure to traditional practices domain; and 0.80 for exclusion domain (Table  2).

**Table 2:** The correlation coefficient of “Infertile women’s exposure to violence determination scale” (IWEVDS)’s subscale and total scale

**Domains**	**Domestic violence**		**Social pressure**		**Punishment domain**		**Exposure to traditional practices**		**Exclusion**		**Total**	
	**r**	**p**	**r**	**p**	**r**	**p**	**r**	**p**	**r**	**p**	**r**	**p**
**Total**	0.91	0.00*	0.88	0.00*	0.91	0.00*	0.76	0.00*	0.85	0.00*	1	0.00*
**Domestic violence**	1		0.76	0.00*	0.81	0.00*	0.57	0.00*	0.72	0.00*	0.91	
**Social pressure**	0.76	0.00*	1		0.75	0.00*	0.60	0.00*	0.75	0.00*	0.88	0.00*
**Punishment domain**	0.81	0.00	0.75	0.00*	1		0.62	0.00*	0.76	0.00*	0.91	0.00*
**Exposure to traditional practices**	0.57	0.00*	0.60	0.00*	0.62	0.00*	1		0.60	0.00*	0.76	0.00*
**Exclusion**	0.72	0.00*	0.75	0.00*	0.76	0.00*	0.60	0.00*	1		0.85	0.00*

*p ≤ 0.01

## Discussion

The scale called “Infertile Women’s Exposure to Violence Determination Scale” indicates high reliability, good content and construct validity.

According to the results of the factor analysis, five sub-scales were determined. In the remaining part of the discussion section, these five sub-scales are discussed separately.

**Domestic violence domain,** which consists of 11 items related to physical, economical, emotional, sexual violence and marital difficulties such as threat of divorce, thinking of getting married to a fertile partner, humiliation, not presenting affection. In literature, there are many studies related to this domain [23-25]. Monga et al. [24] found that the marital adjustment was lower in infertile women than the control group [24]. In a Nigerian study, 97 infertile women reported that they were exposed to domestic violence because of their disability of childbearing [25]. In Leung et al.’s study [23], which was conducted on 500 infertile women, %1.8 of them reported that they experienced violence by their husbands [23]. In a Turkish study [26], 87% of the abused infertile women were threatened with divorce by their husbands. Consequently, domestic violence is a common situation in infertility.

**Social pressure domain,** which consists of 7 items related to social difficulties such as stigma, isolation, humiliation, gossip, being made to feel guilty and disabled by community. The meaning of “child” for a community contains economical, psychological, and social values. A child is thought to be a guarantee for the future and old age. A child is considered to be an important manpower in agriculture-based societies. Having a child sometimes gives a person eligibility and respectability in some cultures. All of these factors lead to more social distress for an infertile couple [5]. The disability of reproduction is perceived as a shameful inability and creates a stigma [12,27].

**Punishment domain,** which consists of several areas (6 items) related to insistence of sexual intercourse, nicknaming, been subject to exhausting housework, not being invited to houses of relatives/neighbours, being charged with inability and forced to own the infertility’s cause. Mothering is an essential role for a woman. Without the opportunity to do so, she is deprived of an important aspect of womanhood [9]. Unisa [11] and Dyer et al. [12] showed that women had got various punishments in their societies [11,12]. Yildizhan et al.’s [26] study showed that 19.5% of the abused women were also abused by their husband’s family [26].

**Exposure to traditional practices domain,** which consists of several areas (4 items). Despite an infertile woman’s unwillingness, forcing her to eat some kind of food which is believed to facilitate conception (item 23), going to places to reverse a spell (item 24), being exposed to curious questions about having a child (item 25), making them tell a lie or give an evasive answer. These kinds of practices are quite common in Turkish society. They appear as a distinct subscale in our study.

**Exclusion domain,** which consists of 3 items: being held responsible/being accused of any random misfortune in life because of being infertile, not being allowed in decision-making mechanisms, being compared with fertile women all the time. According to Goffman’s stigma theory, in pronatalist societies, the validation of social identity is formed by the cultural construction of gender roles linked to reproduction. Hence the concept of being the “other” and being culturally rejected or forced into isolation [9]. It shows that infertile women were exposed to violence by stigma in this subscale.

There was a surprising finding. The aim of the study was to develop a scale which is able to evaluate the exposure to violence not only for women but also for men. But the men have been excluded from the analysis. The reason of this was that the data collected from the men was inconvenient because of their very routine answers. Their answers were very routine because of their prejudgement against the objective of the study. In literature it is reported that in some cultures, a diagnosis of male-factor infertility is socially unacceptable. Because male infertility implies a lack of masculinity and is, therefore, stigmatizing. Many men keep their diagnosis a secret [9]. The underlying reason of their prejudgement might be the thought that it is a clear evidence of their denial of the diagnosis and being vulnerable to the violence because of its negative effects on them. In addition, according to Turkish society’s norms related to patriarchy, the exerter of violence can only be a man, not a woman. So their denial of participation might be related to living in a patriarchal society. Actually, masculinity issues related to infertility are not specific only to Turkish society; apparently, it is global in nature. There are some studies with Greek men [28], Chinese men [29] and Egyptian men [30,31]. In some African cultures, if male-factor infertility is the problem, there is typically significant denial by all parties (husband, wives, and even caregivers) and a lack of treatment. This cultural norm is presumably to protect the male ego and the “superior” role of the male in the society and family [30,32].

When looked through the clinical aspect, it is reported that there is an association between the “level of distress” and the “rate of conception” [2,4,7]. Hence, psychosocial evaluation is as important as medical evaluation. It is possible to scan infertile women for exposure to violence via IWEVDS. In general, domestic violence is often overlooked and most physicians do not routinely screen for domestic violence in infertile women. Routine screening for domestic violence in infertility clinics is necessary to give affected women an opportunity to access appropriate health care and support services. On the other hand, common use of IWEVDS in infertility clinics provides increased sensitivity and awareness of caregivers.

## Conclusion

Briefly, according to the statistical analysis results, it has been determined that Infertile Women’s Exposure to Violence Determination Scale (IWEVDS) indicates high reliability, good content and construct validity.

The IWEVDS is a self-reported scale, with 31 items in five subscales, which takes approximately 10–15 minutes to fill up. It is recommended that caregivers employ the IWEVDS when evaluating infertile couples, because it can be used for routine screening for violence. In the future, the translation and use of the scale in different languages may be useful for other countries with similar traditional practices and hospital settings where lack of a tool for routine screening is felt.

It is recommended that IWEVDS can be used for future studies for psychometric measure on larger sample sizes including multi-centre and community-based. IWEVDS must be examined for validity and reliability when it is issued for community-based studies. Additionally, it is suggested to develop a tool for infertile men for the same objective as this study. I would like to draw your attention to what Dhillon et al. [33] suggested: A man’s true feelings are best derived from interview rather than psychometric data in their study [33].

### Strength of the study

This scale is the first tool to assess violence among infertile women. The scale will make sure that infertile women can be evaluated or determined in regards to violence. It will enable future researches on violence among infertile groups.

In order to generate item pool, besides scanning literature, 16 infertile males and females were interviewed as individual in-depth. The in-depth interviews made valuable contribution to understand the nature of violence as a topic. This methodology can be considered a strength of the study.

### Weakness of the study

The mean weakness of the study is having a small sample size. Although a sample size of 166 people seems theoretically sufficient, a larger sample would be more advisable for a more powerful analysis.

Although there are some screening tools such as Hurt, Insult, Threaten, and Scream” (HITS), “Abuse Assessment Screen” (AAS) and Partner Violence Screen (PVS), none of them are specific to infertility. Unfortunately, due to lack of a similar tool for infertility, external validity could not be conducted. No other violence screening tool that is mentioned above could be precise enough to use for external validity. Moreover these tools are longer than the Infertile Women’s Exposure to Violence Determination Scale. It would not be a good method to use them for external validity.

Although the sample size was adequate with an even distribution of the socio-demographic characteristics of women, the fact that this research was conducted at a single infertility clinic might be considered a limitation of the study. It is recommended to use larger samples for future research. The psychometric measurement must be done on large samples.

Another limitation is that the scale is only for infertile women. At the beginning, it was the plan to develop a tool which is able to objectively evaluate the exposure to violence among not only infertile women but also men. But data regarding men was not convenient for a statistical analysis. Therefore data that belonged to men had to be excluded out of the analysis. The reason for the data of men being inconvenient was their very routine and identical answers. The surprising finding is that, it gives away a great tip to the readers for understanding the cultural reality, which cannot be found through any tool or quantitative study.

The last weakness of the study is that the scale is in Turkish. For other societies, lingual and cultural validity must be done before use.

## Abbreviations

IWEVDS: Infertile women’s exposure to violence determination scale IWEVDS; IVF: *In vitro* fertilization; CVI: Content validity index.

## Competing interests

The author declare that she have no competing interests.

## Authors’ contributions

GO planned the study, collected data, analysed and wrote the final text.

## Authors’ information

GO is an assistant professor dr. at Istanbul Aydin University in the Nursing Department. She is the head of the department. Her PhD. thesis was about infertile couples’ marital relationships and their quality of life. When she was researching for the doctorate, she realized that there was domestic violence among infertile couples. After her PhD. study, she started to research violence among infertile couples. On the other hand, she conducted a study about violence in a shelter in Turkey with qualitative methods.
